# Gas Furnaces and Enamel Fillings

**Published:** 1888-06

**Authors:** William Herbert Rollins


### GAS FURNACES AND ENAMEL FILLINGS.

BY WILLIAM HERBERT ROLLINS.

I claim that what is valuable in the methods and apparatus which Dr. C. H. Land has patented and claims as original, is taken from the results of my investigations, made public property nearly ten years ago. Briefly stated these are :

1st. The discovery of the causes of gasing in firing pottery and porcelain.

2d. The introduction of a current of hot air into the muffle to prevent reduction of the colors, with its attendant results.

3d. The use of pieces of pottery, porcelain and enamel, moulded to accurately fit cavities in teeth.

4th. The invention of a practical baking furnace, using gas, or a mixture of gas and naphtha.

If, however, the members of the dental profession prefer to take licenses from the Land Company for the right to use methods and apparatus that were given them as long ago as 1880, I have no objection. So long as members of the dental profession who patent the results of their researches and work then for their own profit are honored to the highest extent in our power, by being asked to lecture before our schools and before our societies, so long will dentistry remain a trade, and I for one shall be ashamed ever to use my dental degree. Well did Merriam say in memorable words: "What would be thought of Dr. Bigelow, and how would his name go down to history if he had asked or received from his brothers a royalty for each time they performed his operation for stone, or sold his instruments so that they could be withdrawn from competing with those in the market, or patented and received a revenue from them?"

As this is the last time that I shall allude to the origin of gas baking furnaces and crockery fillings, I shall give the data at some length. Prior to 1879, I had devoted much time to firing keramic compounds by gas and naphtha. In the year named I had found out the causes of failure, and had perfected a practical furnace, which has been in constant use from that time until within a year. In 1879 or '80, I read a paper on this subject before "The Society for the Advancement of Oral Science." I exhibited methods of moulding enamel, porcelain and pottery, to accurately fit cavities in teeth, described my furnaces, showed the cause of gasing, explained that this could always be avoided by using an excess of hot air in the muffle, and exhibited my instruments and apparatus. By vote of the society this paper was sent to the *Boston Medical and Surgical Journal.* It was returned by Dr. Hamilton Osgood, who said it was "too technical" for his journal. I then made one more attempt to make the investigations more widely known than they had been by the reading of my paper. I sent the paper to *The London Lancet.* If any one is curious on this subject, I would refer him to that journal for 1880, where he will find that the paper was accepted for publication "in an early number."

In this connection, this abstract from *The Lancet* for March 21, 1885, may be of interest. "Gas Furnaces for Dental Purposes. In reference to criticism by Mr. Fletcher of a paper (by Dr. Rollins) in *The Boston Medical and Surgical Journal,* it is but fair to the latter gentleman to say that, early in 1880, we received from him a paper which not only contained a full account of a gas furnace for baking dental porcelains and artificial teeth, but was also accompanied by a wood cut taken from a photograph of the furnace."

I waited a long time in vain for my paper to appear. Afterward, when I was connected with the *Boston Medical and Surgical Journal,* I made abstracts of this paper, which were published from time to time. "Enamel Fillings" was printed April 26th, 1883. "Gas Furnaces and Causes of Gasing in Firing Pottery and Porcelain" October 23, 1884, and at other times these investigations have been alluded to. These articles were copied into other journals, but I cannot tell which ones, as it is impossible for any one who takes as many journals as I do to preserve them; they would fill his house in a few years. I remember that the two mentioned were reprinted in *Items of Interest.* I am sure of this for two reasons. First, because I was surprised that Editor Welch should have published these as original communications, when, at his request, the editor of the *Boston Medical and Surgical Journal* had loaned him the electrotypes which were used to illustrate the articles. I thought it an unfair return for the courtesy shown to him, and it made a deep impression on my mind. Second, because these articles having met the eye of Thos. Fletcher, he wrote to *Items of Interest* a very unjust criticism of them, saying, among other untrue things, that I must be singularly ignorant to suppose there were any advantages in using hot air in a baking furnace, an arrangement only intended to save fuel in large iron furnaces. He said several other disagreeable things, but I quote this one to show that even this manufacturer did not understand the object of heating the air blast in baking pottery and porcelain, one of these objects being to prevent cooling the muffle by introducing cold air in the proportion required to prevent gasing from the imperfect combustion, which is likely to result if the amount of air is not large enough to more than unite with the gas. As I clearly stated that a slight excess of air should be used, it seems as if one of my reasons for using a hot blast might have been apparent, even to Mr. Fletcher.

I do not claim to have been the first to produce heat enough by gas to bake teeth, but I do claim to have been the first who did so bake teeth, and I was able to do this by reason of my experiments on the causes of gasing in firing pottery and porcelain. I do not claim to have been the first to have filled teeth with crockery, but I do claim that I was the first to mould pieces of pottery, porcelain and enamel, to accurately fit cavities in teeth, and therefore was the originator of the modern method of enamel filling. When I began my investigations, I did not know that there was any furnace in which gas was used that teeth could be baked in, or that pieces of enamel or porcelain had ever been used to fill teeth. I have since learned that the latter method was used before I began to practice, but the grinding of a piece of artificial tooth, some time during one's professional career, to fit a simple hole in a tooth, is quite another thing from the modern method of enamel filling, and does not prevent me from rightly claiming to have originated this.

There is one other point in which Land claims originality, to which I should like to call attention. He claims to have discovered that carbon monoxide will pass through cast iron at baking heat. It seems an insult to the intelligence of the profession for a man to make such a statement as original. Dr. Derby's investigations, more than fifteen years ago, showed that this was true at a much lower heat. Any schoolboy could have told Land this, as it got into the text-books years ago and became a part of schoolboy literature.

There is one part of the Land Furnace which is original with him. This is the introducing of hot nitrogen into the muffle, through a separate pipe, to prevent gasing. A more foolish complication to a good baking furnace could not well have been devised. It is difficult to see why he adopted it, unless it was to have something different from my methods. What is the use of such a complication, when all that is needed is a slight excess of air, which can be carried in with the gas without any separate apparatus?

PLATINUM AND OTHER MOULDS FOR ENAMEL FILLINGS.

Platinum moulds were described by me eight years ago, and I should have nothing to say on this subject now, was it not that everything relating to these methods of mine is being made the subjects of patents by persons who care more for their own private gains than for the good of the profession. On this account, no details of procedure are too petty to put on record, for they may be of use to the profession in preventing the enforcement of unjust patents which may be granted in future.

The deposition of platinum on a non-metallic surface, as that of an impression, is not particularly easy for a beginner, so these simpler ways may be used. Make the copper mould described in previous papers,* and after cleaning the surface as is usual in galvanoplastic operations, stop off the back and deposit the platinum on the copper. When the deposit is thick enough the copper can be dissolved, when the platinum mould is ready for the introduction of the enamel, which can be baked in the mould as soon as dry. Made in this way, the filling is a little smaller than the cavity, but it answers well enough. If one is more particular, he can make a counter mould, and then deposit the platinum on this. Another way is to line the cavity in the tooth with some readily soluble metal, which at the same time is soft. This can easily be done with thin sheet copper, which is laid over the cavity and made to fit it perfectly by means of a wad of cotton and blunt pluggers. Then fill up the hole in the copper with modeling, or other suitable composition, and when this is set remove the copper from the tooth. Or, instead of taking the impression directly from the tooth, a copper mould can first be made as before described, and then the impression in thin metal made from this. In either case, the platinum is to be deposited on the back of this male impression, which, when the platinum is thick enough, is to be dissolved in acid, leaving the platinum mould.

---

* Impression Compound. Take of mastic 100 parts, paraffine 50 parts, black lead 30 parts. Melt and mix together and form into cylinders one quarter of an inch in diameter and one inch long.

For use, prepare the cavity without undercuts, and smear it with a minute quantity of petroleum to prevent adhesion of the modeling compound. Soften only the extreme end of one of the sticks and press it firmly into the cavity. The cold part of the stick will act as a piston, producing a most perfect impression. Make several such impressions of each cavity. Then wrap a fine copper wire around each impression, dip them for a moment into ether and then into black lead, or precipitated silver, brush until there is an even coating and then deposit copper on the surface in the ordinary way, till the coating is thick enough; afterwards, when the impression compound is removed and the mould cleaned, it is ready for use.

Another way is to take the impression according to the method originally described; that is, with a stick of modeling composition, making from this a copper mould about an eighth of an inch in thickness, then from this a die and swaging a platinum plate to fit, thus producing a mould in platinum by swaging. Or make the die in copper, and the counter die in softer metal, thus producing, when the platinum is stamped, a counterpart of the cavity in the tooth, and of the same size inside.

In addition to these, there is the first method I employed, because the most obvious, that of using platinum fitted to the hole in the tooth by means of cotton and a blunt instrument, thus making at once a platinum mould for the baking of the enamel. This method is, however, less perfect than some others which I have given.

Another simple way is to take the impression as just described, deposit a thin layer of copper on it, just enough to make a coherent layer of metal, and then on this copper deposit the platinum till it is the right thickness; afterward, when the copper is dissolved off, we have a platinum mould which is a very little larger than the cavity in the tooth.

Platinum is not an easy metal to work by electricity without practice, so I give one method for this. Dissolve the metal in aqua regia, precipitate with ammonia water, wash the precipitate and dissolve in an aqueous solution of tartrate of soda. Use this solution in the same way that the sulphate of copper solution is used in depositing copper. Other organic acids will answer instead of tartaric, but I usually prefer the one named; nor is soda the only base which can be used.

Better moulds can be made by electricity than in any other way; in fact, the time will come when a small electric plant will become as much a part of a dental laboratory as a vulcanizer now is.

In past years I have shown that better dies can be made by electricity than by the methods in ordinary use. A copper die made by electricity is hard and of the same size as the impression, which it reproduces in the minutest details. That chemically pure amalgams can be more readily made by electricity than in any other way, I showed five years ago. I have also made all gold crowns by this method, without seam and fitting the root perfectly.

These are but a few of the things which we can make electricity do for us. Of some of these others I shall write in another paper.

